# The Hospital. Nursing Section

**Published:** 1905-11-11

**Authors:** 


### The Hospital.

### Nursing Section.

Contributions for this Section of "THE HOSPITAL," should be addressed to the EDITOR, 'THE HOSPITAL" NURSING SECTION. 28 & 29 Southampton Street, Strand, London, W.C.

No. 998.—VOL. XXXIX. SATURDAY, NOVEMBER 11, 1905.

### Notes on News from the Nursing World.

### RED CROSS NURSES CARRYING ARMS.

THE massacre of Jews at Odessa has filled the hospitals in the city with victims. Red Cross carts were used by the Revolutionists for the purpose of carrying ammunition to their comrades. As an illustration of the brutality of the soldiers, it is stated that in some cases they searched women "dressed as Red Cross nurses," and finding firearms on them they stripped and beat them. While condemning this abominable outrage, we think that nothing can justify anyone wearing Red Cross uniform carrying firearms.

### LADY CURZON'S WORK IN INDIA.

PRESIDING for the last time at a meeting of the Dufferin Fund and the Victoria Memorial Scholarships Fund Committees at the Viceregal Lodge, Simla, on October 17, Lady Curzon mentioned that she had been keenly anxious to use their hospitals as training centres. To accomplish this the teaching staff had been so increased and strengthened that there were now employed ten more qualified lady doctors, seven more assistant surgeons, and 337 more hospital assistants and women of the third grade than in 1898; while the number of women under training, apart from *dhais* and nurses employed in the wards of their hospitals, was 476. With respect to the Victoria Fund, which was organised by Lady Curzon herself in 1901, and is training about 160 midwives, she said that in many parts of India the results were admirable. Where they could not train *dhais* in hospitals they sent teachers into the districts, who combined elementary teaching of reading and writing in the vernacular with teaching of hygiene, sanitation, and elementary medical principles. Lady Curzon has done a most valuable work in India, and we hope and believe that the wife of the new Viceroy, Lady Minto, will take it up with vigour where she has left it.

### TORONTO GENERAL HOSPITAL.

THE usual annual gathering in connection with Toronto General Hospital was held last month. After Miss Snively had read her report, showing that there are now 88 nurses in the school of which she is Lady Superintendent, Dr. Bruce Smith presented the certificates and badges, and delivered the address to the graduating class. The Mayor subsequently presented the prizes, the first prize going to Miss Lucy Hurlburt, and the second to Miss Elizabeth M. Laidlaw. The first prize is annually given by Dr. Charles O'Reilly, who has just retired from the post of Medical Superintendent after 30 years' service, and the second in memory of the late Walter S. Lee, who was connected with the Hospital Trust for nearly 30 years. In conjunction with Dr. O'Reilly, he established the Training School for Nurses in 1881, which is now recognised as one of the best in the Dominion.

### VISITING NURSES IN DETROIT.

THE visiting nurse in the United States corresponds to the district nurse in this country rather than to our idea of the visiting nurse. Last month a new Home was opened for the Visiting Nurses' Association of Detroit. The building is of modern style, substantial in character, commodious, comfortable, and fitted with every convenience on thoroughly up-to-date lines. The staff consists of a superintendent and four nurses. A dispensary is attached to the Home, and here each nurse fills the satchel which she carries when she pays her visit. In addition to the usual requisite materials, she takes with her malted milk and beef tablets, though she does not provide food unless it is absolutely necessary. Food, however, can easily be prepared in the kitchen, and clothing may be obtained for destitute patients. The nurses do not attend contagious cases, but if, by accident, they come in contact with them, facilities for disinfection are provided in the basement of the Home. A Detroit lady gave the whole of the funds for the site, building, and equipment of this admirable institution.

### THE PROMOTION QUESTION IN NEW ZEALAND.

THE question of promotion from inside has just arisen at Auckland Hospital, New Zealand. There are twelve sisters attached to the institution, and three vacancies having occurred, it became necessary to decide how they should be filled. The lady superintendent first sent a report to the committee, in which she stated that none of the staff nurses under her was at present eligible for promotion to the rank of sister. The matter was next referred to the chairman of the honorary staff, and, after consultation with his colleagues, he addressed a communication to the chairman of the Hospital Board, in the course of which he practically endorsed the conclusion of the lady superintendent, pointing out that at the present time the junior nurses, whether first, second, or third year, were imperfectly trained. It might have been supposed that in these circumstances the Hospital Board would have adopted a proposal on the part of the chairman to follow the advice of the honorary staff. But instead of doing so, they determined, by a majority of 5 to 3, to suggest to the staff that out of the eight staff nurses three sisters be appointed, and to ask, if the honorary staff did not agree, for "clear reasons" from them for disagreeing. It appears to us that the clearest possible reasons for not choosing the sisters from inside have already been given, and we hope to hear later that the course proposed by the lady superintendent, supported by the honorary medical staff, has been adopted. If nurses are fully trained and qualified, occasional promotion from inside is to be welcomed. But it cannot be for the advantage of any hospital that probationers should be converted into sisters before the matron and the medical staff consider that they are eligible. This is pushing the principle of promotion from inside too far.

### THE HOUSING QUESTION IN EAST LONDON.

The Mile End Guardians, like the War Office, have to solve the question of housing their nurses. At present the accommodation is of a very unsatisfactory and primitive character, and it is generally admitted that something must be done. The majority of the Guardians are in favour of erecting a new home at a cost of about £8,000; but a minority contend that the necessary provision can be made for the nurses by adapting existing buildings for the purpose. A meeting of ratepayers has been held in opposition to the proposed expenditure, and we entirely sympathise with the view that their money ought not to be spent in superfluities. But a spacious and comfortable home for the Infirmary nurses is not a superfluity, and we believe that the course advocated in this case by the majority of the Guardians is likely to be more economical in the end than the alternative plan of patching up the old buildings. There are nearly fifty nurses employed in the wards of Mile End Infirmary. Both in the interests of the sick poor and of the nurses themselves, it is most desirable that the latter should be properly housed, and that no costly compromise should be allowed to carry the day.

### AN IRISH MATRON ON PAUPER NURSING.

The Lady Superintendent of the Protestant Infirmary at Dublin, and the Rev. Mother in charge of the Roman Catholic Infirmary, both affirm that the scheme submitted by Dr. Caleb Powell to the Guardians of the North Dublin Workhouse Infirmary merits support. Following the decision of the Guardians themselves in favour of the scheme, this significant agreement means, we hope, the early establishment of a training school on the same lines as the great Poor Law Infirmaries on this side of St. George's Channel. Miss West, in expressing her views on the subject, states that "large workhouse infirmaries make better training schools than the small general hospitals," but whatever difference of opinion may obtain on this point, her declaration that "the system of employing able-bodied inmates of workhouses to assist in the nursing is a greater evil than appears at first sight," will carry conviction to all who know anything about pauper help. We trust that its doom has been sounded in North Dublin.

### EXAMINATION BY THE CENTRAL MIDWIVES BOARD.

The number of candidates examined by the Central Midwives Board at the examination in October was 463, the number passed was 352, and the percentage of failures 24. The training schools most conspicuously represented by the successful candidates were the General Lying-in Hospital; Clapham Maternity Hospital; Queen Charlotte's Hospital; the Maternity Charity, Plaistow; Guy's Trained Nurses' Institution; City of London Lying-in Hospital; British Lying-in Hospital; East End Mothers' Home; Glasgow Maternity Hospital; Manchester Maternity Hospital; Liverpool Lying-in Hospital; and Jessop Hospital, Sheffield.

### NURSES WITH NICE MANNERS.

There was an advertisement in a daily paper this week from a nursing agency for a "Lady nurse, with about a year's training, for a private hospital of 20 beds. Must have nice manners and be willing to commence work at once. Good salary given. Can have further training if necessary." We fear that it it the employment of "lady nurses with nice manners" and very little training, in nursing homes and private hospitals, which has done so much to bring them into evil repute, and there is no probability of matters improving until these institutions are registered and open to periodical inspection.

### THE VETO OF THE LOCAL GOVERNMENT BOARD.

The Penzance Guardians recently selected a superintendent and an assistant nurse, and in due course notified this to the Local Government Board, who promptly vetoed the appointments. In their letter they informed the Guardians that the proposed superintendent nurse did not seem to have received any skilled instruction in nursing, or to have had any such experience as would fit her for the post of head nurse. With regard to the other nurse, the Local Government Board pointed out that apparently she was entirely without the practical experience in nursing contemplated by Article II. of the Nursing in Workhouses Order, 1897. The letter also stated that, as the workhouse was some distance from the medical officer's residence, it was imperative that a person who was competent in midwifery should be at the workhouse. The Board requested that both the appointments should receive further consideration by the Guardians. Mr. Preston Thomas, the inspector for the South-Western District, who was present when the unwelcome communication was received, told the Guardians that the Local Government Board were surprised at their desire to appoint as head nurse a person who had no certificates and in whose testimonials there was nothing to show she was competent in midwifery. Thereupon the Guardians at once decided to choose a nurse who has the requisite qualifications, while the proposed superintendent nurse is to be her assistant.

### THE ROYAL BRITISH NURSES' ASSOCIATION IN THE TRANSVAAL.

In reply to the protest which has been made in a Johannesburg paper against the proposed acceptance for registration by the Medical Council of the Transvaal of the certificate granted by the Royal British Nurses' Association, on the ground that it is granted to women who have worked in hospitals for one or two years and to some with fever training only, the Secretary of the Association, Miss Annie J. Hobbs, sends us the regulations of that body. The first of these is to the effect that "Candidates for registration must produce proof that they have been engaged in training in a hospital or infirmary recognised by the Board, and containing not less than forty beds, for a period of three years, of which period the whole or a portion of one year may have been devoted to training in one or more special hospitals recognised by the Board as schools for instruction in fever or other special branches of nursing." We note, however, with regard to the controversy which is still proceeding in the Transvaal papers, that a distinct case is cited of a nurse at the Johannesburg Hospital who is a member of the Association holding only a two years' certificate from the Derby County Hospital. It is also stated that there are two members of the Association in the Transvaal, one with fever training only and the other with a two years' certificate from Kimberley. The matter seems to be one for inquiry in London, but there must be a mistake about Derby, which has no County Hospital. The training at the Derbyshire Royal Infirmary is for four years.

### THE MIDWIVES AND ASTON UNION INFIRMARY.

There is considerable consternation at Aston Workhouse Infirmary in consequence of the refusal of the Central Midwives Board to approve the application for the recognition of the institution as a training school for midwives. The decision, which was discussed at the last meeting of the Aston Guardians, is based upon the report of the Inspector sent to Aston, who has advised the Board that the structural and general conditions of the Infirmary are unsatisfactory. The view of the Guardians is that the facilities offered in the matter of obstetrical cases are almost equal to any in the Midlands, and they fear that the refusal of the Midwives Board to recognise the Infirmary as a training school will seriously affect the supply of nurses. We are afraid that it may have that effect, but the Central Midwives Board naturally acts upon the recommendations of the inspectors it employs, so long as it has confidence in their judgment.

### QUEEN ALEXANDRA'S MILITARY NURSING SERVICE.

We are officially informed of the following changes in Queen Alexandra's Imperial Military Nursing Service: Miss G. A. Howe, staff nurse, has been posted to Cambridge Hospital, Aldershot, and Miss C. W. Jones, staff nurse, to Queen Alexandra Military Hospital, Millbank, S.W. Miss G. M. Smith, staff nurse, has been transferred to Egypt from Woolwich; Miss E. M. Perkins, staff nurse, to Egypt from Netley; and Miss A. Fitz-Gerald, sister, has been directed to hold herself in readiness for service abroad. Miss B. Rennie, sister, has resigned her appointment.

### PRESENTATION TO MISS VERNET.

The new Matron of the Middlesex Hospital received, before her departure from the National Hospital for the Paralysed and Epileptic, substantial tokens of the respect and regard in which she is held in that institution. The nursing staff, past and present, presented her with a silver tea service; the chaplain, architect, secretary's staff, resident medical officers, and dispensers, with a silver kettle and tray; and the domestic staff, male and female, with a revolving bookstand, silver tea knives, and a hymn-book. Miss Vernet has now entered upon her duties at the Middlesex Hospital.

### SUNDERLAND DISTRICT NURSES AND THE PENSION FUND.

The Committee of the Sunderland District Nursing Association have decided to take out policies in the Royal National Pension Fund for Nurses for all nurses who have been in the service of the Association for five years and to maintain these policies whilst the nurses remain in the service of the Association.

### THE NURSES' MISSIONARY LEAGUE.

We understand that the Nurses' Missionary Union will in future be known as the Nurses' Missionary League. There is a department of the Young Women's Christian Association called the "Nurses' Union," which has been in existence for several years, and as some of its members and workers seem to find the two titles somewhat confusing, they have asked that the younger association should change its name. This request has been readily complied with.

### GUARDIANS AND OUTDOOR NURSING.

The Croydon Guardians were recently invited to instruct the Infirmary Visiting Committee to submit to the Board a list of patients in the infirmary whom the medical superintendent is able to certify as capable of being treated and nursed in their respective homes. The author of the proposal said that it was "merely a resolution preliminary to asking the Board to appoint district nurses." But it transpired that the Infirmary Committee had ascertained that morning that there were forty-eight cases fit for nursing outside, in the event of proper accommodation and nurses being available. It was suggested, however, that before any steps were taken an estimate of the expenses of the scheme for district nursing should be submitted. The Guardians have since received a report from the Infirmary Committee recommending that the relieving officer should be requested to inquire whether the homes from which these and subsequent cases were sent were suitable for nursing outside.

### A WARNING TO NURSES AT PORTS.

We are asked to warn nurses at sea-side resorts and ports against helping a young man who professes to have lost his ship. He states that he is related to a nurse, and tries to borrow money for his journey home. He looks about 20, is a little over 5 feet in height, has dark hair, and is very pale. It appears that several nurses have already been victimised by this individual, but an attempt he made to obtain money in Kent suggested the inquiries which prove that he is an impostor.

### OUR CHRISTMAS DISTRIBUTION.

We have received contributions to our Christmas distribution from Miss S. F. Francis, Burton-on-Trent; Miss Knights, Tufnell Park, N.; and A Private Nurse, Nottingham. All parcels should be addressed to the Editor, 28 and 29 Southampton Street, Strand, London, W.C., with "Clothing Distribution" written on the outside.

### SHORT ITEM.

Among the successful candidates at the examination of the Clapham School of Midwifery, which we mentioned last week, were three probationers who are in training at the Workhouse Infirmary at Bagthorpe, Nottingham.

### The Nursing Outlook.

"From magnanimity, all fear above;
From nobler recompense, above applause,
Which owes to man's short outlook all its charm."

### THE WALTHAM SYSTEM.

Last week we published a detailed account of the Waltham Training School for Nurses, which Dr. Worcester established at Waltham, Massachusetts, U.S.A., in 1885. That account proves to demonstration that the Waltham School has rendered a great service to nursing by insisting upon a thorough training in the many details of the work, and an exhaustive test of the personal qualities of each probationer, especially in regard to her motherly, tender, and thoughtful care for each individual patient committed to her charge. There can be no doubt that the attention given at Waltham to the first year's preparatory course, and the thoroughness with which instruction is afforded in all the various departments which it includes, marks a new and important departure, the necessity for which has become recognised, and may consequently cause a similar course to be established in connection with every efficient nurses' training school. With the exception of mental nursing the Waltham system is entitled to claim that it was the first to provide a complete course of training for every one of its probationers.

A personal inspection of the school and its working has convinced us that it would be well if every educated girl, on the completion of her education, could enter and pass through the first year's preparatory course. By so doing she would render herself efficient in the duties of the head of a household to an extent not otherwise possible of attainment elsewhere at present. It is undoubtedly true that no one can satisfactorily supervise the work of others unless he or she is actually acquainted with what constitutes a full day's work in every department of duty which passes under review. The comfort of every home is largely dependent upon the smooth, economical, and efficient working of the whole household economy, and we know nothing better calculated to secure this result than a preparatory course similar to that in force at the Waltham School.

Although the first purpose of this School is to supply the nursing needs of the community, it has become apparent that there are great educational advantages in the methods of training employed. The Waltham system, after all, is but a return to the original plan pursued at Kaiserwerth, which produced Florence Nightingale, and it is in effect a necessary protest to the utilitarian custom of subordinating training schools to hospitals. This custom is in reality a very modern invention, which has not succeeded in producing ideal nurses, because it has led probationers to regard interesting cases as the beginning and end of their work, and has so made them incapable in many instances of properly discharging their duties to the really ill who may be suffering from an acute aggravation of some common ailment, although these very diseases, in fact, affect the majority of the population.

By following up the work of the Waltham advanced probationers in other institutions we ascertained that they have failed in regard to the actual handling of medical and surgical cases, for the reason that the teaching has been devoted mainly to thorough training in everything which makes the nurse useful and efficient in a private household, but has failed in part to equip the student with a complete mastery of the technique of hospital nursing. The instructors have, however, supplied the deficiencies in their nurses which are so apparent in many turned out by the larger training schools. But in securing this desirable result, they have failed, so far, to recognise the importance and necessity of retaining the probationers in the hospital at Waltham continuously for at least twelve months. The difficulty has been caused by insufficient funds to place the probationers when in the hospital under constant trained supervision and instruction in the wards, and to thus secure that they shall have obtained a thorough mastery of the technical side of hospital nursing in all its aspects. In a word, the Waltham probationers require the same careful supervision and the enforcement of the same thoroughness in regard to their hospital training which the teachers in this school have so successfully organised and enforced in regard to every detail of the first year's training, the home nursing, the obstetric side of nursing, and the care of chronic and dying patients.

We therefore congratulate Dr. Worcester upon having secured the services of Miss Pringle, as head of the Waltham Hospital Training School, and we hope that he may soon obtain the $2,000 a year extra revenue which is needed to defray the cost of providing graduate nurse instructors in every department of the hospital, to take charge of and to train thoroughly the Waltham probationers in this branch of their work, in which at present they are found to be not so efficient as some probationers trained by the large training schools. When this change has been effected we believe that the Waltham nurse will be everywhere popular, and that those portions of the Waltham system which do not yet find a place in the curriculum of the large training schools will be adopted by these schools in self-defence. Otherwise they may lose their reputation, and so fail to secure a sufficient number of probationers for training in their schools.

### Abdominal Surgery.

By Harold Burrows, M.B., F.R.C.S., Assistant Surgeon to the Bolingbroke Hospital.

### SYMPTOMS IN ABDOMINAL DISEASE.

(*Continued from page* 56.)

### Abdominal Expression.

The remark is often made, when the diagnosis of a case is under discussion, that the patient has an "abdominal expression." The term sounds sufficiently vague, and yet when present the appearance which it indicates is so characteristic as to be of great value. In fact it is not infrequently possible for the experienced observer to tell almost to a certainty whether he has or has not an acute abdominal case to deal with, merely by glancing at the patient. The latter, if he is acutely ill with an abdominal lesion, and especially if peritonitis is present, lies on his back, perhaps with his knees drawn up, and his face unmistakably betokens acute pain and anxiety. The eyebrows are raised, the eyes though wide open appear sunken, with dark rings round them, the corners of the mouth are depressed, the cheeks are hollow, and the chin is thrust forward. These and other indefinable details go to make the "abdominal expression," which once seen and carried in the memory will nearly always be a sign of the greatest significance when the presence of serious mischief in the abdomen is suspected. The phenomenon is often as well marked in babies a few weeks or months old as in adults. But while an adult almost invariably lies on his back if he has serious abdominal trouble, children under the same condition often lie curled up on one side, and I have seen a child suffering from fæcal vomiting due to intestinal obstruction, and so ill as to have an imperceptible pulse, sitting up in bed. It is well to bear this difference in mind.

### The Pulse.

Pallor and thirst do not require any detailed consideration; it is enough to mention that they are common accompaniments of acute abdominal trouble.

The frequency and character of the pulse are important features in nearly every case. In peritonitis the pulse is usually more frequent than 100 beats per minute, and may be 150 or 160 or more. Exceptions do occur, chiefly in men and old patients of both sexes, and especially when large doses of opium or other sedative have been given. As peritonitis progresses the pulse tends to be increased in frequency, and this increase is a valuable sign.

In a case of appendicitis, for example, it is sometimes doubtful whether the inflammation will remain localised to the immediate neighbourhood of the appendix or will gradually spread over a large part of the peritoneum. If the trouble remains localised it is often advisable to postpone operating; indeed an operation may not be needed at all. But if the inflammation spreads an immediate laparotomy is almost certain to be necessary, and if so the less delay there is in performing it the better. But the exigencies of his work will probably prevent the medical attendant from remaining constantly with the patient, and so he will be compelled to rely upon the nurse to watch for any evil signs in his absence, and to make an immediate report to him if any such signs occur. The chief one for which she will be required to look out is an increasing frequency of the pulse. In order to detect any change she will have to count the pulse at frequent intervals, say every half-hour, or every hour, and to record her observations. If the pulse suddenly becomes much more frequent, or shows a progressive increase at each occasion, even if the increase be slight, the nurse should at once report the change to the surgeon, as it is quite likely that an immediate operation will be required.

Care must be taken to cause as little disturbance as possible to the patient when counting his pulse, otherwise correct estimations will not be made. Patients who have peritonitis are more than usually susceptible to slight perturbations, and a little jolting of the bed and disarrangement of the bed-clothes will readily increase their pulse frequency, even by so much as twenty beats per minute. The nurse should be sure her hands are warm, and she should feel for the patient's wrist in the gentlest manner, without dragging his hand from beneath the bedclothes and without chattering to him meanwhile. To the inexperienced these warnings may seem too trifling to be regarded with serious attention; if so, it is because to see their value the sick man's spectacles are needed.

The character of the pulse is nearly as important as its frequency in the diagnosis of abdominal inflammation. In the early stages of peritonitis the pulse is frequent, small, and hard. This is often called a "peritoneal pulse," and is a common and easily recognised accompaniment of early peritonitis. As the illness progresses and the patient becomes worse, the character of the pulse changes; while the frequency is still high and perhaps increasing, from being hard the pulse becomes softer and softer until it is imperceptible.

### The Excretions.

With regard to the excretions it is only necessary to mention that specimens should always be kept on the first occasion, as their analysis may supply vital information. Subsequently the nurse will be guided by the doctor's instructions, or by the appearance of some obvious abnormality.

Unhappily this advice is too often ignored, with results most disastrous to the patient and to the nurse's reputation. There may be no other way to make a correct diagnosis than by analysis of the excreta for blood, pus, and other pathological evidences, and on a correct diagnosis the patient's life perhaps will depend.

### Distension: Visible Peristalsis.

Distension of the abdomen is a prominent feature of many abdominal cases, and is one of the most frequent and troublesome complications occurring after operations. The nurse should always be on the look-out for this symptom.

She should also be able to recognise visible peristalsis when present. The term peristalsis is applied to the movements which the stomach and intestines perform in order to propel their contents onward along the bowel. Normally, nothing can be seen of these movements by inspection of the abdomen. But if obstruction to the flow of intestinal contents is present, the bowel immediately above the obstacle becomes distended, and at the same time contracts more vigorously than usual in order to force its contents past the obstruction. Owing to the distension of the bowel and to its excessive activity, the peristaltic movements can often be distinguished by looking at the surface of the abdomen, especially in thin people. In those who are stout the peristaltic movements may be obscured on account of the thickness of the abdominal wall. Peristalsis may be audible as well as visible, and as it is a help to the surgeon in some instances to know that peristaltic movements have been observed, the nurse should endeavour to provide trustworthy information on this point.

### Temperature and Respirations.

The patient's temperature is not of great value as a sign of abdominal trouble. As in other regions of the body, an inflammatory process in the abdomen commonly causes a rise of temperature. But this is not so invariably; indeed, there may be a marked fall. The correct interpretation of the temperature chart is only possible in the light of considerable experience. The nurse's duty as regards the patient's temperature does not extend beyond carefully taking and recording it at regular intervals and reporting any sudden and considerable change, either a drop or a rise, as soon as it is discovered.

The respirations should be counted also at regular intervals, and charted. Reference has been made previously to the fact that acute abdominal pain may be an early manifestation of pneumonia, and in such a case, unless the greatest care is taken, laparotomy may be performed in the mistaken belief that there is serious mischief in the abdomen. One of the ways by which this error may be avoided is by paying attention to the rate of respiration, which is usually considerably higher in chest complaints than in other illnesses. For this and other reasons an accurate chart is of great value in all abdominal cases.

### The Nurses' Clinic.

### THE NURSING OF ANTHRAX.

One Sunday afternoon a short time ago, just as our patients' visitors were leaving the hospital, and we were busily getting a ward ready for the service which is held here every Sunday, a closed carriage drew up at the front door. A few minutes afterwards a servant brought me a message from Sister —— asking me to admit the patient who was waiting outside. On opening the door of the carriage I could see at once that it was a serious case. The patient—a young man of 28 years of age—was looking desperately ill, and evidently was suffering intense pain. His wife was with him, and said that her husband was a farmer, and that a few days before he had scratched his thumb with a rusty nail in the stable, but went on with his ordinary occupations, which included the skinning of a dead cow! Four days afterwards he began to feel pain in the arm, but took very little notice of it until it commenced to swell and become much inflamed. Then he showed it to a doctor, who told him to come to the hospital at once, for the arm by that time had doubled its size, and black marks had begun to appear. I helped the man to get out of the carriage; he would not let me put him into a carrying-chair, as he thought it would hurt him less to walk; so with the help of two nurses he managed to reach the ward. But as soon as he got there he sank down on the bed quite exhausted, and groaning with pain. We waited a few minutes for him to recover somewhat before proceeding to undress and get him into bed. In the meantime we gave him a drink of hot milk, filled hot-water bottles, and warmed a blanket for him, as he seemed very cold and shivery. His temperature on admission was 102°, but we heard afterwards that it had been 105° before he left home.

As soon as we had him comfortably settled in bed we took off the linen rags which were bound round his right arm, and found that it was tremendously swollen and inflamed, and that the swelling was rapidly extending over the shoulder and down the right side towards the back.

On the thumb was a small punctured wound caused by the rusty nail. On the upper arm was a black, circular patch of pustules, about the size of a five shilling piece, and a smaller one on the forearm, about two inches below the other, the size of a sixpence, and a similar patch in the middle of the forehead about the same size.

Ours is only a small hospital of 26 beds, and we have no resident surgeon, so a message was at once sent off to the "surgeon for the week" to tell him about the new patient.

In a very short time the doctor arrived, and after thoroughly examining the man, and hearing his history, his diagnosis was "anthrax," and said that the only thing to do for him was to freely remove the black patches at once. So another doctor was sent for to administer an anæsthetic, and we were told to get everything that would be required in the ward, as the patient was not to be moved from the bed.

Screens were placed round the bed while all the preparations for operation were being made. Compresses of 1-20 carbolic were applied to the three patches and a simple enema given before the other doctor arrived.

When the man was told that an operation was necessary he begged the doctor not to give him anything "to send him to sleep"—he was willing to bear any pain if only it could be done without an anæsthetic! However, when the doctor told him that it would be best for him to have it, as it was bound to be a painful operation, he consented to try it, but after three or four inhalations he absolutely refused to continue and simply entreated the doctor to cut the places out at once, faithfully promising not to move or utter a sound if only he need not be chloroformed. So, of course, there was no help for it, it had to be done, and one patch after the other was quickly removed and placed at once into a bowl of lotion. We had been warned beforehand to be very careful of our hands. The poor man bravely kept his word; he bore it all without flinching, nor did he utter a sound, and even said after it was all over "That he would rather bear it all over again than have that 'horrible stuff!'"

Each of the wounds was well swabbed with "strong mixture," composed of liq. hyd. perchlor. 1-500, acid carbolic 1-20, and then boracic fomentations applied, which were renewed every four hours.

The patient had a restless night after the operation, a good deal of pain and hardly any sleep. The next morning the temperature was still up, and when the doctor came he

THE NURSES' CLINIC—*Continued.*

told us to telegraph to London for a box of Sclavo's anti-anthrax serum, which was sent to us by passenger train that same day.

In the evening a 10 c.c. subcutaneous injection was given in the abdomen. There was no rise of temperature after it, and the swelling began to go down, and the wounds were sloughing. On the fourth day the patient complained of abdominal pain. A second 10 c.c. injection of serum was given. The next day he began to feel better and had very little pain, except when the dressings were changed. The temperature came down and he seemed much better.

For the first week he had no solid food, but was kept on milk, eggs, and beef-tea; and quinine, grs. 5, was given every four hours, and whisky half an ounce every three hours. At the end of the week he was allowed to get up, and was then put on to fish and chicken. He gained strength rapidly, all the swelling disappeared, and the temperature remained normal. He had rather a bad five minutes one day, when the doctor moved the elbow joint; it was beginning to get stiff through being kept up in the sling; but after that all went well, and we gave him a weight to carry for a few minutes at a time several times a day. We continued the boracic fomentations until the sloughs had separated, then we changed them for a boracic ointment dressing, and the patient was allowed to go home, taking some ointment with him, having been in hospital only twenty days.

### Nursing Small-pox in the Australian Bush.

BY AN ENGLISH NURSE.

We had been travelling all over the country in search of a spot that would prove suitable for my father to get strong after a serious illness. One place after another we tried, but the heat only brought on dysentery, until one of the doctors in Melbourne made a suggestion. "Why not go up into the Bush," he said; "right out of the populated parts of Melbourne, which is only a modern London?"

So, by easy stages, stopping a few days in any places which were nicer than the others, we found ourselves in a small village in the Bush country. When I say that we were fifty miles from a railway station, and that there was only one store, from which you could buy anything from a pound of butter to a costume, or from lamp oil to bootlaces, and that the said store was supplied from the nearest small town once a month only, by a waggon and team, it will be realised how isolated we were. There were about 120 inhabitants, counting children, in the village, and when we arrived some of them very kindly made room for us in their homestead, until houses which were being built were finished. All were made of wood, and built raised from the ground—high enough for anyone to crawl under, and all the rooms were on one floor; no stairs, but sometimes with a verandah all round, and over which sweet peas, honeysuckle, convolvulus and other lovely creeping-plants were trained. Not only did these plants give a nice shade, and yield a delightful scent, but unfortunately the mosquitoes loved to congregate there to such an extent that those who ventured too near sometimes got badly bitten. There was a Wesleyan minister in the place, who had come of his own accord some three years before, and at whose instigation a tiny mission-room had been built of corrugated iron, close to a running stream which was big enough to be a tributary of some river, and round this little mission-room the yellow wattle grew, and the weeping willows dipped their lower branches in the stream.

It had been a very hot summer, and one day a man, with a pack of billy-tins and cans of all sorts on his back, came wearily up the verandah steps offering them for sale. The "billy" is a mug-shaped can used for lading water, a most useful thing on a wash-day, so we bought some from him, and when I paid him I noticed a queer red eruption all over his hands, and, upon looking more closely at him, I saw the same thing on his face. His eyelids were swollen, and there was a general puffy look all over his face. Thinking the heat, as well as the long tramp which the man had undoubtedly had, might have brought out some eczema on him, we were glad to let him go, and it was nearly six weeks later before it was my lot to see him again.

Three weeks had nearly gone by when our minister called to see father, and said his wife was feeling so very poorly, that mother said that I should go down and see her.

When I did go she seemed hot and feverish, with red spots thickly out on her face and neck. I asked her if she had a thermometer, but beyond one on the wall, which registered the room temperature at 120°, there was no other in the house. So I ran back to ours for one. We always carried two clinicals about with us in case of one being broken, on account of father being so often ill.

When I took the poor woman's temperature it was 104°, so I undressed her and got her off the couch in the sitting-room and to bed. I tried hard to persuade her to have the children's beds made up in another room, but she insisted upon keeping the six months' old baby with her.

I went home sorely perturbed in my mind and said to mother that I thought the minister's wife had chicken-pox very badly, and that as I had been touching her I should like to go back and do things for her till she got better.

I was nearly seventeen at this time, so, with mother's consent, I packed together a few things, as I knew I was to stay till my patient was better, and, chicken-pox being infectious, I could not keep running home for things.

During the night the poor woman tossed and moaned all the time, attempting frequently to get up.

I had to call her husband to take the baby out of the room towards morning, when she was too delirious to notice that the mite had gone, and early on the following day he decided to go to the nearest town for a doctor. Getting the only "buggy" available, he drove off, and it was quite late at night before they came back in the doctor's trap.

The doctor looked very serious indeed when he came in and saw the condition of the patient, and asked if I was the only nurse to be got in the place—a young, inexperienced girl. "Are you aware," he said, turning to me, "that this is confluent small-pox of the worst type, requiring strict isolation and the most watchful nursing, day and night?"

Of course I was not aware of anything of the kind, but I begged to be allowed to stay and nurse the patient, and said I would obey to the letter his orders.

"But you cannot do it all by yourself," he replied. Here the husband interposed and said that if some neighbour would take the two children he would isolate himself and share the nursing with me. So that was how it was arranged.

The doctor made up a white ointment with which we painted the patient's poor face, her eyelids were so swollen that the eyes were invisible, and later on we had to break the formation of scabs round the mouth to insert the nozzle of a feeder with nourishment. The doctor could only come once in every four or five days, and on his second visit three more people, all in different houses, were down with the disease.

He vaccinated as many of the helpers and the patients as he was able to with the limited amount of lymph at his command. My own arm did not "take," but in some of the other cases the result was quite satisfactory.

There being some difficulty about isolation in these places, the minister proposed to the doctor that, as he was unable to take service as usual, it would be a good plan to turn the little mission-room into a temporary hospital, taking out the chairs, and removing patients, bed and bedding, feeding utensils, etc., from each house into it. This was done the following day, and the evening found us with our four patients settled in the little mission-room. It was light and fairly well ventilated, and the scent of the honeysuckle and sweet-peas came through the windows.

A woman, who took in sewing in the village, volunteered to help me with the nursing at night, and a relation of the minister's wife helped him during the day. At the end of the week there were nine "cases" in our little temporary hospital; the women on one side of the hall and the men on the other—a long curtain run through rings on a line dividing the place down the centre. Before we had been there three weeks there were twenty-four patients, and at the end of the month thirty-two; that, I am grateful to say, was the total.

By this time those who had been taken ill first were convalescent, and able to help with the acute cases. Three patients died, one the sempstress, who had so kindly volunteered to help, she having evidently been vaccinated too late, as her arm was very bad at the time she was stricken down. The minister buried the dead tied up in their bedsheets, just as they were, in a bit of fallow waste land close to the stream, and a weeping willow waved a solemn requiem over them as the slight night breeze soughed through its branches.

Tents were pitched at the rear of the hall for the helpers and for the convalescents, and, with only this slight accommodation, we managed somehow. We carried out the doctor's orders as far as possible, but it was very hard on us, untrained as we were, to have to rely on our own scanty knowledge in an emergency. We made little frames of wire and covered them with white mosquito net and fitted them with tapes over the patient's faces during delirium to prevent the perpetual scratching and rubbing, which has such fearfully disfiguring results, and I must say that our patients did us credit as far as regarded their appearance afterwards.

It was hard work, though, keeping such delirious patients in bed, without getting them rubbed. One day the cow-keeper's boy came to the door of the hall in great excitement. He had been four miles away from the village searching for a cow which had strayed, and he said that in a huddled-up heap under a hedge was a man, and when he went near him he was quite dead, and a horrible sight, and by his side was a bundle of cans and billy-tins!

Like an electric shock this news affected me.

It was the man with the billy-tins, then, who had brought the scourge to our village!

I begged to be allowed to accompany the minister to the spot, and although he tried to dissuade me I got my way.

But when we arrived there and I saw the object, I felt almost sorry I had come, for there was but little of the face left. The cow-keeper's boy and the minister, who had brought spades, dug the man's grave where he lay.

One incident happened that afternoon which impressed that awful scene in the brilliant sunshine for ever on my memory. The grave was nearly dug when a queer chuckling noise seemed to come from the direction of the heap which had once been a man, and in fear we started and turned round. A laughing jackass had perched himself on the hedge over the body, and was making that queer sound which only those birds can make, and which I had never heard before. It stayed there and laughed and laughed till the body was covered with the earth, and as the minister slowly said the prayer of consignment, the bird's laughter grew shriller, and it was not until we started to leave the plague-stricken spot that the laughing jackass too spread his wings and flew away, as though he, too, was glad to leave the scene.

Just ten weeks had elapsed from the time I had left my mother to go and nurse the minister's wife with what I then thought was chicken-pox, before I presented myself, all freshly disinfected, at our little temporary home again.

It was very good to be back again, and mother has never ceased to be thankful that I came through the ordeal without contracting the disease. We did not stay long in the village after that, but came back into a more populated part of Melbourne, and it was just two years later that I entered a fever hospital as a probationer, which would have been the last place in the world I should have gone to, if I had not had that experience of nursing during the outbreak of small-pox in the Bush.

### Impressions of a Swiss Clinique.

Far away in a city overlooking one of the Swiss lakes stands the Clinique. It comprises two large houses, connected by a glass corridor. One is entirely devoted to patients, also part of the other, the rest being reserved for the Professor's private apartments.

The Professor, of course, is the "Chief." He is a typical professor—grey hair, keen eyes, prompt, decisive. Even British stolidity cannot hide anything from those eyes. Calm, firm, sure hands and touch; no wavering, no shirking, no getting out of what must be borne for the patient. Yet all the time you are being treated his hand and eye are bracing you to endurance.

> . . . "the kind eyes
> Soothing yet nerving you."

The Clinique is for treatment of ear, throat, and nose diseases, on which, of course, the Professor is a specialist. So many and varied are his patients that an assistant and nurse are necessary to help him through the day's work. The nurse is hardly what the English term conveys. She is more a maid of all work. But she has certainly had some training, for she understands the battery, assists at operations, and evidently knows the names of lotions and instruments, besides being able to give a hypodermic injection, which she does with evident distress, always politely asking you to look the other way and saying she knows one "must think her barbarous, but Monsieur le Professeur commanded her." She is really a smart nurse, but just a trifle untidy in appearance. It is a pity she wears no uniform, for the fresh daintiness and quiet dignity of the English nurse seems wanting, although she is every bit as capable in her work.

The Professor begins work at 8 A.M., when he sees non-residents. The rest of the morning, sometimes until 2 P.M., is spent with us. On several afternoons during the week he has consultations and hospital visits, and lectures on other evenings.

If it is true that genius is just the "infinite capacity for taking pains" perhaps the Professor owes his large share of it to that, for he does a tremendous amount of work himself; including small things which so often fall to the nurse's share as insignificant. He even gives the hypodermics himself, except when he is unavoidably detained at hospital.

My bedroom is snow-white; so dainty and cheerful; cosy armchair, large window, polished wood floor, which shines

IMPRESSIONS OF A SWISS CLINIQUE—*continued.*

radiantly. The bell can be touched or electric light switched on without moving one's position in bed. I really think if one must be ill, a Swiss Clinique is a delightful place in which to do the deed.

It is very interesting to sit in the waiting-room at 10 A.M. and watch the various patients. It is such a contrast to the English specialist's waiting-room, where there is usually an ominous silence and it would be bad form to smile, although *Punch* is often lying on the table.

Here the patients are somewhat demonstrative and very sympathetic. They do not in the least mind showing their feelings when the maid announces that the Professor awaits them.

There is a pretty little Russian lady who, when she is called, looks very demure, gives her shawl a nervous shake, gets up languidly, gives the maid an appealing look, and finally goes, leaving behind a sympathetic audience.

There is a Swiss boy who assumes a debonair manner and always makes a point of saying something cheerful to his sister before he goes.

There is a charming Spanish girl, several other Russians, Hungarians, Italians, Parisians, Dutch, and so on *ad lib.*

After we have seen the Professor and rested lunch is next on the programme. It is quite a gay time. Everyone appears to have recovered their spirits; the Swiss boy chats to the Spanish girl, who says the wittiest things in the weakest of voices. The Russian ladies discuss the menu, others the war and the papers. Everyone is talking.

The table is always nicely laid, bright with fruit and flowers, and the cooking, of course, is excellent.

The Clinique is superintended by a charming Swiss gentlewoman. She is married, speaks four languages well, and looks after all our wants and fads in an astonishingly capable manner. She is a great reader, and there is hardly a patient who will not find a book they can read in her room.

I got a stolen glance at the operation-room yesterday. I was passing the door when I met the Swiss boy, who said: "Mademoiselle, do you know what this is?" touching the door. I knew, and felt quite creepy, which was doubtless ridiculous. But, oh! when one's the patient instead of the nurse how different one's sense of proportion becomes! So I barely answered, "If it is locked beware; don't you know the English history of Bluebeard?" However, I was not to escape. "See, it is unlocked," was the answer, "come and look."

The room greatly resembled our operation-room—tiled floor, operating-table, instrument-case, bowls (enamel), and dressing-boxes. The light, perhaps, was not as good as we should like.

The cheerfulness of all the patients strikes me forcibly. Of course the majority quite recover, but the treatment—a great deal of it is electricity—is often long and tedious, and sometimes most painful. Then there are the few inevitable chronics, and I do think deafness is most depressing, but even they seem philosophical. For example, one said to me in the course of conversation, "No! the Professor will not promise me anything, but I feel sure what he can't do no one else can, so I shall go home and make the best of it." The Professor inspires this infinite faith in all his patients.

We have a delightfully large sitting-room, and leading out from it is a large, long, broad balcony, so nicely arranged with tables, lounge chairs, and cushions. Every morning after treatment I go and sit there. Below stretches the lake, beyond rise the snow-clad eternal hills, evoking all kinds of comforting thoughts. After the whirr of the battery and the ache in one's head it is so soothing to sit there in that peaceful atmosphere.

If, as I am sure most nurses would agree, hope is half the cure of any disease, I think the balcony, with its position and inviting chairs, is just a subtle touch of the Professor's genius.

### Appointments.

[No charge is made for announcements under this head, and we are always glad to receive and publish appointments. The information, to insure accuracy, should be sent from the nurses themselves, and we cannot undertake to correct official announcements which may happen to be inaccurate. It is essential that in all cases the school of training should be given.]

Carnarvonshire and Anglesey Infirmary.—Miss Annie L. Burkill has been appointed charge nurse. She was trained at the Royal Infirmary, Manchester, where she has since been charge nurse.

Grantham Hospital.—Miss G. Fitzgerald has been appointed night sister. She was trained at Manchester Royal Infirmary, where she has since been staff nurse.

Great Yarmouth Hospital.—Miss M. L. Bond has been appointed staff nurse. She was trained at Bethnal Green Infirmary, and has since been staff nurse at the Samaritan Free Hospital for Women in London.

Gretton Cottage Hospital.—Miss Annie Burrows has been appointed matron. She was trained at the Royal Infirmary, Manchester, where she has since been staff nurse. She has subsequently been head nurse at Dorset County Hospital; head nurse at the Northern Fever Hospital, Winchmore Hill, London; nurse matron at Butleigh Cottage Hospital; temporary night sister and private nurse at Southport Infirmary. She holds the City of London Lying-in Hospital's certificate.

Guy's Hospital.—Miss Mildred Brown and Miss Mabel Chitlock have been appointed respectively surgical night sister and second house sister. Miss Brown was trained at Guy's Hospital, and has since been on the private nursing staff and probationary sister. Miss Chitlock was trained at Guy's Hospital, and has since been sister at Charing Cross Hospital.

Kasr-el-Ainy Hospital, Cairo.—Miss Minnie E. R. Barr has been appointed sister. She was trained at St. Thomas's Hospital, London. She has since been sister in the Royal Sea Bathing Hospital, Margate, and the Royal Infirmary, Bristol. She holds the certificate of the Central Midwives Board.

Stirling District Asylum, Larbert.—Miss Jean McGrigor has been appointed matron. She was trained in general nursing at the Victoria Infirmary, Glasgow, and in mental training at Larbert Asylum, where she has been on the staff for nearly two years as assistant matron.

The Royal Hospital, Chelsea.—Miss Elsie Satchwell has been appointed matron. She was trained as to general nursing at the London Hospital, and has held important posts in several institutions, including the matronship of a private hospital. In April 1904 she was appointed to the matronship of the male side at the Stirling District Asylum, Larbert, N.B., and a few months later was promoted to be matron of the whole asylum, which post she vacates in order to take up the matronship at Chelsea.

Throat Hospital, Golden Square, London.—Miss M. Oakes has been appointed sister. She was trained at Southwark Infirmary, East Dulwich. She has since been staff-nurse at the Liverpool Stanley Hospital, and sister at the Stockton and Thornaby Hospital, Stockton-on-Tees. She has also undertaken holiday duties at St. Peter's Hospital for Stone, Covent Garden, and private nursing in London.

Walsall Workhouse Infirmary.—Miss B. M. Milk has been appointed superintendent nurse and Miss N. F. Brash charge nurse. Miss Milk was trained at Nottingham Union Infirmary, and has since been charge nurse at Walsall Union Infirmary. She holds the certificate of the Central Midwives Board. Miss Brash was trained at Nottingham Union Infirmary, and has also been nurse at Glasgow Fever Hospital.

### Incidents in a Nurse's Life.

Contributions to this column are invited.

### THE NIGHT BEFORE THE "FINAL."

A merry tea-table gathering composed of about twenty nurses. The home sister was absent, so the girls were presiding over their own table.

"Oh dear!" said "Gordy," a bright little nurse, mockingly pulling a long face. "How shall we all feel this time to-morrow? I, for one, shall be weeping my eyes out; I'm such a duffer!"

"Now, then, Gordy!" was the general disclaimer, "don't be affected; you know quite well that you're the cleverest of the whole lot of us."

"Yes," chimed in a shrill little voice; "I only wish I knew half as much. I don't know a thing that the greenest 'pro.' doesn't know, and I haven't studied for the past three months. I guess, too, I can't cram now, for my poor head is ticking like a clock, and if I try to imbibe any more knowledge I'm right sure it'll burst."

"Stick to your guns, Yankee," was the encouraging advice. "You'll 'come through' all right."

'Mid the ensuing hubbub of conversation "Yankee" whispered to her neighbour: "Look here, 'Gordy.' What do you say to a mock exam. to-night? We might be lucky enough to hit on some of the real questions. My room will just about hold us all."

"Jolly!" assented "Gordy" eagerly. "Good idea."

"Attention, ladies, please. We're going to have a mock exam. to-night in 'Yankee's' room. Who's coming?"

Several "I wills" broke in immediately.

"Any coffee or tea provided?" queried somebody.

"Oh, yes," was the general chorus at once, "let's have tea and cake. We shall work ever so much better after it. A cup of tea does wake one up, you know."

"But," said "Yankee," "dare we risk the tea? You know what it says in the 'rule book' about 'food in the bedrooms,' and matron is so down on the 'spirit-stove' business. We're pretty sure to get 'bowled.'"

"Nonsense!" cried the daring "Gordy." "If you're nervous about it, 'Yankee,' we'll have the meeting in my room. Let us say my room, girls—number twenty—tea and cake at 9.15 p.m."

Room twenty, at nine o'clock, presented a very strange appearance for a respectable hospital nurse's bedroom. On the marble washstand was a grand array of assorted utensils: an old black tea-pot with the spout chipped and the lid broken, and replaced with a much cracked saucer; the bowl of a soap-dish, washed thoroughly, did duty as a sugar-basin; whilst the milk (purloined from a ward kitchen) found a place in an old medicine bottle.

A bubbling, gurgling noise from the vicinity of the fire-place indicated that the water was boiling over the spirit-stove. Then a cautious little tap sounded on the door, followed shortly by a second; and, one after another, the nurses, each supplied with their own cup or glass, crept into the room.

Some found a place on the bed, others squatted on the floor, whilst three turned a chair lengthways to act as a form, the remainder busying themselves piling up biscuits and buns on a clean folded towel in lieu of a plate.

The good things were rapidly disappearing, when a sharp tap came on the door. Scared glances were flashed from one to another, and with one accord dresses and aprons were fussily spread out, and glasses and cups quickly secreted under them, whilst an awed and breathless silence ensued.

The tap resounded once more, and "Gordy," whilst noisily calling out, "Who's there?" quickly flopped down on the stove standing on the hearth, pushing the saucepan under the bed.

A suppressed and knowing giggle was borne on the air, and in walked "Yankee," full of apologies for being so late, locking the door behind her.

"Oh, you dreadful girl!" cried "Gordy." "Why did you give us such a fright? We'd given up expecting you, and were afraid that it was matron, and that we were caught red-handed. Well, here's your tea; help yourself to cake."

Hardly were the words uttered when there was another and more imperative knock at the door, and the handle turned sharply but ineffectively.

"Heavens!" breathed "Gordy." "It's all up this time. We're caught. Keep still, for goodness' sake!" And, rising stealthily, she seized a pile of books from the shelf and rapidly handed one to each girl without uttering a word.

Immediately every nurse opened her book and pretended to be reading very intently.

Again came the knock—this time with some irritability—and a voice—Matron's—cried, "Open the door at once, Nurse!"

"Gordy" jumped quickly up, as if startled, and, uttering many apologies for being so deeply engrossed in her book, flung the door wide open on a number of nurses, apparently very busy studying.

"Eh! let me see. This is your room, Nurse Gordon, is it not?"

"Yes, Matron."

"And you are just having a little recapitulation to make sure of yourselves for to-morrow? . . . Well, it won't do you any harm; but don't sit up reading too late, or you'll not do justice to yourselves when the time comes."

One poor girl, who had hastily grabbed hold of the milk bottle, had unfortunately managed somehow to turn the bottle upside down, with the result that she was soon sitting in a veritable puddle of milk.

There was no carpet, and a white stream was gaily coursing on towards Matron's feet. The poor girl hardly knew what to do for the best. If she appeared restless Matron would naturally glance towards her and catch sight of the milk; whilst, if she kept still, the unchecked stream would reach Matron in a few seconds.

Hardly had the seriousness of this threatened danger been realised by the nurses when a maid came hurriedly up with a message that the "house surgeon would like to speak to Matron immediately," and, with a cheery "Good night" to the nurses and a wish for their success on the morrow, the good soul departed in answer to the surgeon's request.

The much relieved nurses gave no further thought to study that night, but trooped thankfully off to their beds, congratulating each other on their lucky escape and remarking that "Matron was not so keen as she used to be."

A week had passed since the much dreaded "Final." The nurses were jubilant, having all passed exceedingly well and beaten preceding records. But just now, however, they were much exercised about a summons to Matron's office, which had been given to every nurse who had entered for the examination. "Nurses," said matron when they were all congregated in the room, "you will no doubt remember that on the eve of your recent examination I came to a certain nurse's room and found that she had apparently invited you all to meet for the purpose of studying. You may, however, be surprised to hear that I was not so blind as you thought. The tea-party arrangement was known to me right from the time of its proposal, for I was inspecting the stock in the pantry, which adjoins the mess-room, when nurse suggested it. I was loth, however, to censure and upset you at such a time, when your whole mental energy and concentrated thought would be so much needed. But I warn you all now that if such disobedience is ever repeated I will not condone it, but will report the matter further.

"Now, nurses, before dismissing you I should like to add that I am proud of each one of you, and just because of that pride I want to feel I can also trust you, one and all, to do the right thing always towards your hospital, your matron, and yourselves."

### Everybody's Opinion.

[Correspondence on all subjects is invited, but we cannot in any way be responsible for the opinions expressed by our correspondents. No communication can be entertained if the name and address of the correspondent are not given as a guarantee of good faith, but not necessarily for publication. All correspondents should write on one side of the paper only.]

### THE CONFERENCE OF QUEEN'S SUPERINTENDENTS.

"W. B." writes from Manchester: There will probably be a large meeting of Queen's Superintendents at the Conference to be held in London on the 29th inst. Would it not be possible for the advocates of registration to arrange for a discussion to take place on either that or the following day? A great many of the visitors, I am sure, would be only too pleased to learn more about it.

### PRESENTS AT CHRISTMAS.

"E. A. L." writes: For years I was on the staff of a local hospital. Thus having to collect subscriptions at Christmas most fully do I agree with "M. R. N. P. F." that the gifts being practically compulsory became a burden. May I suggest as a way out of the difficulty that a box should be placed on a shelf in the dining-room some little time before? Then those nurses who wished could give what they could afford.

"A. B. C." writes: The giving of these is, as your correspondents say, rather a tax. It seems to me that it only wants someone to have the courage of their opinion in order to stop it. In the hospital where I had my first sister's appointment I at once informed the nurses in my ward that I did not accept presents. This, of course, led to a certain amount of unpopularity among the sisters; but in a few years' time it became general in that hospital. Now that I am a matron I refuse all presents of any kind from the hospital staff. At Christmas we make a general collection for the servants, and each gives what she can—sisters 4s. or a little more, and nurses 2s. Then each servant has a nice gift on Christmas morning, and the only other expense is a few pence for the postman. This all give willingly.

"The Superintendent of a North Country Nursing Institute" writes: May I, as one of the matrons, say how thankful many of us would be not to have a Christmas present—not out of any ungracious spirit, for one values all tokens of goodwill from one's nurses, but a hearty "Happy Christmas, Matron," brings sometimes more pleasure than many valuable gifts, especially when one thinks that perhaps several amongst the staff would have had to forego sending a present to one of their "home people," rather than seem mean or poor in not subscribing to the gift given to an official. However much they may respect and love their matron, a feeling of aggravation must crop up. But alas for custom! One does one's best, intimating in the most careful way that one would much prefer their greetings to the most valuable of gifts. Some one among the staff will always raise the subject, and, like a flock of sheep, all follow. A gift on taking up an appointment or leaving, or after some years spent with them, is a different matter. My nurses are dear, nice women. I like them, and I think they truly like me, but I do so wish that they would not tax themselves for me.

### THE USE OF WALLETS.

"Anti-wallet" writes: I was much surprised to notice in your columns a paragraph devoted to bringing a new wallet before the notice of nurses. It is termed "The Aseptic Wallet." It may be a slight improvement on the old wallet, but that is about all that can be said for it. Wallets are distinctly undesirable, and to the modern, well-trained nurse an impossibility. The instruments are exposed to dust, and may come in contact with anything else while the nurse moves about. Is there any surgeon or properly trained nurse who would consider instruments ready for use, taken from any wallet? Surely not! They must first be sterilised; then, what is the idea of the nurse carrying them about? I am pleased to belong to "a certain class of nurse" who would consider it wrong to use, or advocate the use of wallets in any form. Matrons and others in authority might do much good by prohibiting the use of such things. In conclusion let me hope that wallets will soon be numbered with "Sairey Gamps" and other undesirables of the past.

[The objection to the wallet is in the abuse, not in the use.—Ed. The Hospital.]

### THE PENSION FUND IN TIME OF SICKNESS.

Miss C. M. Löhr, Matron of the Cottage Hospital, Potters Bar, writes: Through the medium of your paper, which has so wide a circulation in the nursing profession, I should so like to bring to the notice of my sister nurses the great benefit which the Royal National Pension Fund is to nurses, and specially in the time of sickness. The benefit it has personally been to me makes me feel that it is only right to bring the notice of the fund to others, who may also benefit by it too. It is now some eight years ago since I joined the fund, and during that time I have had long weeks of illness, not only once, but two or three times, and I have received the greatest kindness, consideration, and courtesy, while the financial help from the sick-pay fund has been of the greatest service through these breakdowns. I would urge all nurses to join and to take out sick-pay assurances. We all know the anxiety caused by a long illness, and the drain it is on the resources of a nurse—anxiety which often retards convalescence. The comfort, then, to those who can look forward to 10s. or £1 a week, as the case may be, is very great. Moreover, we know that it is not a charity, but our own that is coming back to us, which has been so carefully laid by and taken care of by the fund. When I joined the fund I was accepted for five years, and now since my last medical examination I hold a sick assurance policy, which remains good until I take my pension. I believe that nurses object to this second medical examination, but surely it cannot be considered a hardship, for it is a precaution in our favour and to protect our fund from risks. So again may I urge nurses to consider the matter, and to join this mutual benefit club, so that in times of illness they may enjoy the benefit of it, as I have done?

### PRIVATE NURSES IN THE TRANSVAAL.

"A Transvaal Nurse," under date of October 15, writes: There has been much said in the *Transvaal Leader* about the scarcity of the private trained nurse. We promptly wrote to the editor, and begged him not to induce any more nurses to come out nor in any way to add to the ranks of those already here. It is all very well from a sentimental point of view, but better, far better, have an army of trained (or untrained) charwomen, more suited to the needs of the South African public. There is scarcely a house in which it is fitting a patient should be nursed; it is really better and wider hospital accommodation that is needed. The majority of the cases, if they cannot be moved, should be district cases, where a nurse is not called on to be in residence. It is amusing to see how the public always looks upon a nurse as a compendium of all the domestic virtues, and in nearly all houses she is called upon not only to cater and cook for her patient, but for the whole family, not to mention innumerable other household duties which fall to her share. Now, those of us who have not a natural turn for cooking, etc., know that our hospital experience will never teach us—few of us ever entered a kitchen. The Kaffirs are certainly the most wretched servants on the face of the earth. In the Transvaal they are the worst of all in South Africa. They are the only natives in the world who want white man's food, all others providing, cooking, and preferring their own. Therefore a white "Missis" spends half her time in cooking and serving food for these creatures. This, coupled with their general want of intelligence, robs them of half their usefulness. In Natal and Rhodesia an attempt is made to induce them to eat their native mealie pap, but they always rebel if they see other food. In the Transvaal the Kaffir houseboy is paid £3 a month and his food. Nurses are often tempted by the apparently high fees to come out here, but the majority of the people are of a different and rougher class to those we nurse at home; and the pay being high, the cases are very short, as they cannot afford to keep a nurse long. Hence, one is not really repaid for the extra cost of living. The vile, unnourishing food, too, is enough to drive one out of the country. There has been a good deal of talk about the Royal British Nurses' Association, and there is a lady consul at present in Johannesburg for the purpose of enrolling nurses; but, as a nurse in writing to the *Transvaal Leader* says, "Delightful as it is to have the Princess Christian as President, one fails to see how the status of the fully-trained nurse will be improved, as many of those who have joined have not received their three years' certificate."

### Presentations.

Walsall Union Infirmary.—On Friday an interesting ceremony took place at the Walsall Workhouse, when Miss Loach, superintendent nurse, was presented with a handsome gold bracelet, subscribed for by the whole of the staff on the occasion of her leaving to be married. Dr. G. Martin Fox, medical officer, made the presentation and alluded to the cordial feeling existing between the officials. On their behalf he wished Miss Loach long life and happiness in her married state. The Chairman of the Board, in endorsing the remarks of Dr. Fox, congratulated Miss Loach on the performance of her duties—first as probationer, next as charge, and finally as superintendent nurse; her whole service under the Board extending over a period of six years. The Master remarked that the matron and himself were very sorry to lose her, as she had always worked with them in a pleasant and amicable manner, thus enabling the establishment to be carried on without the slightest friction. The superintendent nurse suitably replied, after which the remainder of the evening was spent in social enjoyment.

### Where to Go.

A series of lectures and demonstration of massage applied to the Cure of Wasting Limbs, by Miss E. Grace Riant, County County lecturer upon health and hygiene at the Latymer Road Mission, Blechynden Road, Notting Hill, W., Tuesday, November 14, 21, 28, December 5, at 3 p.m.

### Wants and Workers.

Will any district nurse or sister working among the poorest classes in London send their address to Nurse Bloomer, 40 Wilton Road, Bexhill, who will forward them, carriage paid, a parcel of used clothes for small children?

### Queen Victoria's Jubilee Institute for Nurses.

Her Majesty Queen Alexandra has been graciously pleased to approve the appointment of the following to be Queen's Nurses, to date October 1, 1905:

### ENGLAND.

| Name | District Training at | Serving at |
|---|---|---|
| Lucy MacPherson | Bermondsey | Crook |
| Dena Leather | Birmingham (Newhall Street) | Birmingham (Newhall Street) |
| Gertrude Maude Hardy | Blackburn | Blackburn |
| Edith Emily Tolman | ,, | ,, |
| Eva Margaret Hunter | Bloomsbury | Darwen |
| Margaret Katharine Lea | ,, | Gloucester |
| Barbara Lendrum | ,, | Willington |
| Ada Parry | ,, | St. Helen's and Sea View, Isle of Wight |
| Leonora O'Connell | Bolton | Caldervale |
| Florence Louise Wolverson Hemming | Brighton | Willington |
| Honor Margaret Neale | ,, | Sheerness |
| Harriett Maud Westcott | ,, | Somerset County Nursing Association |
| Margaret Warn | Cardiff | Truro |
| Minnie Harding Close | Chelsea | Brixton |
| Jessie Lachlan | ,, | King's Walden Bury |
| Gertrude Evelyn Moore | Dublin (St. Lawrence's) | Warrington |
| Edith Katharine Clifford | East London (Cable Street) | East London (Cable Street) |
| Isabel Mary Eacott | Gloucester | Newark-on-Trent |
| Helen Maud Graham | ,, | Gloucester |
| Grace Helen Vaughan | Hammersmith | Hammersmith |
| Hilda Beatrice Young | ,, | East Malling |
| Edith Caroline Birch | Liverpool (Central Home) | Liverpool (Central Home) |
| Elizabeth Rose Donaldson Lapsley | Liverpool (Derby Lane Home) | Tunbridge Wells |
| Jane Williams | ,, | Liverpool (Derby Lane Home) |
| Emily Hoskyn | Liverpool (Kirkdale Road) | Hebden Bridge |
| Elizabeth Burnett | Liverpool (Shaw Street) | Liverpool (Shaw Street) |
| Bridget O'Connor | Manchester (Harpurhey) | Manchester (Harpurhey) |
| Annie Spriggs | Oxford | Oxford |
| Alice Louisa Miles | Paddington | Brixton |
| Margaret Hellings Webber | Portsmouth | Witley |
| Minnie McLean | Rochdale | Rochdale |
| Mary Jane Greenhough | Salford | Salford |
| Ruth Wood | ,, | Wisbech |
| Elizabeth Alexandra Fraser | Shoreditch | Crook |
| Florence Juanita Fyers | ,, | Rottingdean |
| Helen Hicks | ,, | Shelford |
| Elizabeth Hirons | ,, | Bath |
| Alice Beatrice King | ,, | Longtown |
| Florence Kearney | Southampton | New Malden |
| Jane Johnston Hadden | Sunderland | Sunderland |
| Sabina Goddard | Walworth | New Malden |
| Mary Isabel Upton | Westminster | Crowborough |
| Emma Frost | | Guildford |

### WALES.

| | | |
|---|---|---|
| Elizabeth Richards | Cardiff | Towyn |
| Elizabeth Anne Roberts | Dublin (St. Patrick's Home) | Ystalyfera |
| Emily Wynne | Liverpool (Central Home) | Llandidloes |
| Margaret Parry | Walworth | Halkyn |

### IRELAND.

| | | |
|---|---|---|
| Maggie McErlean | St. Lawrence's Home, Dublin | Ardee |
| Harriet Sanderson Athya | St. Patrick's Home, Dublin | Dublin |
| Margaret Park Cross | ,, | ,, |
| Hilda May Macoun | ,, | ,, |

### SCOTLAND.

| | | |
|---|---|---|
| Cecilia Mary Jane Begg | Edinburgh | Kingsbarns and Crail |
| Mary Gow Core | ,, | Alexandria |
| Janey Gray | ,, | Kelty |
| Annabella Jack | ,, | Speanbridge |
| Jessie Christian Johnston | ,, | Paisley |
| Emily Grace Moir | ,, | Newport-on-Tay |
| Annie Macintosh Tulloch | ,, | Rothesay |
| Agnes Sutherland | Dundee | Dundee |
| Jessie Young Macnaughten | Glasgow | Glasgow |
| Isabella Thomson Muir | ,, | ,, |

### Notes and Queries.

**REGULATIONS.**

**The Editor is always willing to answer in this column, without any fee, all reasonable questions, as soon as possible.**

**But the following rules must be carefully observed.**

1. **Every communication must be accompanied by the name and address of the writer.**
2. **The question must always bear upon nursing, directly or indirectly.**

**If an answer is required by letter a fee of half-a-crown must be enclosed with the note containing the inquiry.**

*School of Massage.*

(36) I should be grateful for some information about the School of Massage at —— Hill, S.W., and enclose stamped addressed envelope.—*M. A. F.*

The lady who conducts the establishment to which you refer—which is in a pleasant position—is a properly qualified masseuse, but, of course, we can say nothing from experience as to her powers of instruction. Inquirers are again reminded that it is quite useless to send a stamped addressed envelope. Replies are never sent by post unless a fee of 2s. 6d. is enclosed.

*Daily Work.*

(37) I am a trained nurse, general, maternity, and with mental experience. Can you advise me how I can get daily work in Sheffield or Nottinghamshire?—*A Nurse.*

Advertise in the local papers, and make yourself known to the medical men.

*Instruction in Elementary Nursing.*

(38) Can you tell me if there is anywhere in Plymouth where I could take lessons for the St. John's Ambulance course, or a course of lessons in nursing?—*L. M.*

As to the first, write to the local secretary of the St. John's Ambulance Association, Plymouth; and as to the second, to the Matron, South Devon and East Cornwall Hospital, Plymouth.

*Free Homes or Flats.*

(39) Can you tell me whether there are residential homes or flats in London or its neighbourhood where gentlewomen possessing a small annual income (over £40 and under £100, I believe) may be accommodated rent free? I am inquiring on behalf of a nurse, now elderly, who through an injury received while working is incapacitated from further nursing, and has only her pension—not quite due—to fall back upon, and has no friends with whom she could live.—*C. R.*

Write for "Homes and Hospitals for the Benefit of Gentlewomen." You will probably find something suitable for the case you mention. Obtainable from the Scientific Press, post free 1s.

*Naval or Military Service.*

(40) I should be greatly indebted if you could tell me how to obtain the information necessary to enter the naval or military service.—*Nurse A. W.*

For full particulars of Queen Alexandra's Royal Naval Nursing Service, write to the Director-General, Medical Department of the Navy, The Admiralty, 18 Victoria Street, S.W.; and for particulars of Queen Alexandra's Imperial Military Nursing Service write to the Secretary, War Office, 63 Victoria Street, S.W.

*Sanitary Institute.*

(41) Which is the best book to obtain information to pass the examination of the Royal Sanitary Institute? How often is the examination? Must one go to London for it?—*Welsh Girl.*

Write for information to the Secretary, the Sanitary Institute, Margaret Street, S.W.

*Probationer.*

(42) I should be very much obliged if you could let me know if I can from Paris seek a place as probationer for January in a hospital in London, and where could I apply?—*T. L.*

If you send to the Scientific Press, Limited, 28 and 29 Southampton Street, Strand, London, W.C., for the book "How to become a Nurse," you would find many suitable hospitals where you might apply for a vacancy; but nearly all matrons require a personal interview before accepting a probationer.

**Handbooks for Nurses.**

| | Post Free. |
|---|---|
| "How to Become a Nurse: How and Where to Train." | 2s. 4d. |
| "Nursing: its Theory and Practice." (Lewis.) ... | 3s. 6d. |
| "Nurses' Pronouncing Dictionary of Medical Terms." ... | 2s. 0d. |
| "Complete Handbook of Midwifery." (Watson.) ... | 6s. 4d. |
| "Preparation for Operation in Private Houses." ... | 0s. 6d. |

Of all booksellers or of the Scientific Press, Limited, 28 & 29 Southampton Street, Strand, London, W.C.

### For Reading to the Sick.

THE DISTANT LAND.

THE land beyond the sea!
When will life's task be o'er?
When shall we reach that soft blue shore,
O'er the dark strait whose billows foam and roar?
When shall we come to thee,
Calm land beyond the sea?

The land beyond the sea!
When will our toil be done?
Slowfooted years! More swiftly run
Into the gold of that unsetting sun!
Homesick we are for thee,
Calm land beyond the sea!

The land beyond the sea!
Sweet is thine endless rest;
But sweeter far that Father's breast
Upon thy shore eternally possessed.
For Jesus reigns o'er thee,
Calm land beyond the sea!

*Faber.*

When obedience and faith are made perfect, it may be that knowledge and explanation shall be given.

And I do not mind now, Mother. When the sunshine goes, and the wet comes, and the river looks dark, and the sky black, I think about the Unknown Land. . . . This is not our Rest! The river is rushing forwards, the clouds are hurrying onwards; the winds are sweeping past, because here is not their Rest. Ask the river, ask the clouds, ask the winds where they go to:—Another Land! Ask the great sun, as he descends away out of sight, where he goes to:—Another Land! And when the appointed time shall come, let us also arise and go hence.—*Parables from Nature.*

Go on patiently, my child. Each day, as it passes, carries away so much of our trial, and brings us so much nearer the haven where we would be.

Life seems long to those whose lot is a tearful one, but how short it will seem when the blessed day comes in which the Lord will comfort those that are His! Then for the first time we shall really know what those words mean, "Blessed are they that mourn, for they shall be comforted." But a little while, and we shall have done with tears for ever, and rest in His bosom who is the fulness of joy, reunited to all whom we loved here in His holy love.—*A Dominican Artist.*

So when the days of joy are past,
And life's enchantment o'er,
When we have bowed to sorrow's blast,
And hope is bright no more;
There still are mercies full and free
Mixed in the cup of woes,
And where the mourner cannot see,
In faith he onward goes.

